# Improving spliced alignment by modeling splice sites with deep learning

**Published:** 2025-06-15

**Authors:** Siying Yang, Neng Huang, Heng Li

**Affiliations:** 1 Department of Biomedical Informatics, Harvard Medical School, 10 Shattuck St, Boston, MA 02215, USA; 2 Department of Data Science, Dana-Farber Cancer Institute, 450 Brookline Ave, Boston, MA 02215, USA; 3 Broad Insitute of MIT and Harvard, 415 Main St, Cambridge, MA 02142, USA

## Abstract

**Motivation:**

Spliced alignment refers to the alignment of messenger RNA (mRNA) or protein sequences to eukaryotic genomes. It plays a critical role in gene annotation and the study of gene functions. Accurate spliced alignment demands sophisticated modeling of splice sites, but current aligners use simple models, which may affect their accuracy given dissimilar sequences.

**Results:**

We implemented minisplice to learn splice signals with a one-dimensional convolutional neural network (1D-CNN) and trained a model with 7,026 parameters for vertebrate and insect genomes. It captures conserved splice signals across phyla and reveals GC-rich introns specific to mammals and birds. We used this model to estimate the empirical splicing probability for every GT and AG in genomes, and modified minimap2 and miniprot to leverage pre-computed splicing probability during alignment. Evaluation on human long-read RNA-seq data and cross-species protein datasets showed our method greatly improves the junction accuracy especially for noisy long RNA-seq reads and proteins of distant homology.

**Availability and implementation:**

https://github.com/lh3/minisplice

## 1. Introduction

RNA splicing is the process of removing introns from precursor mRNAs (pre-mRNAs). It is widespread in eukaryotic genomes (Keren et al., 2010). In human, for example, each protein-coding gene contains 9.4 introns on average; >98% of introns start with GT on the genome (or more precisely GU on the pre-mRNA) and >99% end with AG (Sibley et al., 2016). On the other hand, there are hundreds of millions of di-nucleotide GT or AG in the human genome. Only ∼0.1% of them are real splice sites. Identifying real splice sites, which is required for gene annotation, is challenging due to the low signal-to-noise ratio.

To annotate splice sites and genes in a new genome, we can perform RNA sequencing (RNA-seq) and align mRNA sequences to the target genome. This approach does not work well for genes lowly expressed in sequenced tissues. A complementary strategy is to align mRNA or protein sequences from other species to the target genome. Spliced alignment through introns is essential in both cases.

**Fig. 1.**
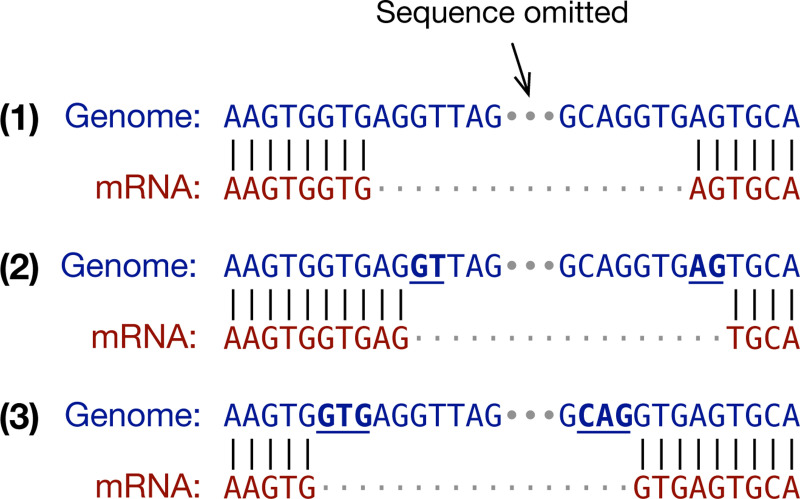
Ambiguity in spliced alignment. The same genome-mRNA sequence pair can be aligned differently without mismatches or gaps.

It is important to look for splice signals during spliced alignment as the residue alignment around a splice site can be ambiguous. For example, the three alignments in Fig. 1 are equally good if we ignore splice signals. However, as the putative intron in alignment (1) does not match the GT..AG signal, it is unlikely to be real. While both (2) and (3) match the signal, alignment (3) is more probable because it fits the splice consensus GTR..YAG better (Irimia and Roy, 2008; Iwata and Gotoh, 2011), where “R” stands for an A or a G base and “Y” for C or T. In this toy example, the query sequence matches the reference perfectly in all three cases. On real data, spliced aligners may introduce extra mismatches and gaps to reach splice sites. The splice model has a major influence on the final alignment especially for diverged seqeences when aligners need to choose between multiple similarly scored alignments around splice junctions.

Position weight matrix (PWM) is a classical method for modeling splice signals (Staden, 1984). It however does not perform well because it cannot capture dependencies between positions (Burge and Karlin, 1997) or model regulatory motifs such as branch points that do not have fixed positions. Many models have been developed to overcome the limitation of PWM (Capitanchik et al., 2025). In recent years, deep learning is gaining attraction and has been shown to outperform traditional methods (Zhang et al., 2018; Du et al., 2018; Albaradei et al., 2020). Early deep learning models are small with only a few 1D-CNN layers. Later models are larger, composed of residual blocks (Jaganathan et al., 2019; Zeng and Li, 2022; Xu et al., 2024; Chao et al., 2024) or transformer blocks (You et al., 2024; Chen et al., 2024). It is also possible to fine tune genomic large-language models for splice site prediction (Nguyen et al., 2023; Dalla-Torre et al., 2025; Brixi et al., 2025). Developed for general purposes, large-language models are computationally demanding and may be overkill if we just use them to predict splice sites.

At the same time, Helixer (Holst et al., 2023) and Tiberius (Gabriel et al., 2024) combined Hidden Markov Models (HMM) and deep-learning models for *ab initio* gene prediction and achieved high accuracy. They however do not report alternative isoforms. Alignment-based methods remain the state of art for gene annotation.

While qualified spliced aligners all look for the GT..AG splice signal, they model additional flanking sequences differently. Intra-species mRNA-to-genome aligners such as BLAT (Kent, 2002), GMAP (Wu and Watanabe, 2005) and Splign (Kapustin et al., 2008) often do not model extra sequences beyond GT..AG because alignment itself provides strong evidence and ambiguity shown in Fig 1 is rare. Minimap2 (Li, 2018) prefers the GTR..YAG consensus (Irimia and Roy, 2008). This helps to improve the alignment of noisy long RNA-seq reads. Protein-to-genome aligners tend to employ better models due to more ambiguous alignment given distant homologs. Miniprot (Li, 2023) considers rarer GC..AG and AT..AC splice sites and optionally prioritizes on the G|GTR..YNYAG consensus common in vertebrate and insect (Iwata and Gotoh, 2011), where “|” indicates splice boundaries. Exonerate (Slater and Birney, 2005), Spaln (Gotoh, 2008; Iwata and Gotoh, 2012; Gotoh, 2024) and the original GeneWise (Birney et al., 2004) use PWM. GeneSeqer (Usuka and Brendel, 2000) and GenomeThreader (Gremme et al., 2005) apply more advanced models (Brendel and Kleffe, 1998; Brendel et al., 2004). Deep learning models have been applied to refining splice sites as a post-processing step (Chao et al., 2024; Xia et al., 2023) but have not been integrated into spliced aligners.

In this article, we introduce minisplice, a command-line tool implemented in C, that learns splice signals and scores candidate splice sites with a small 1D-CNN model. We have modified minimap2 and miniprot to take the splice scores as input for improved spliced alignment. Importantly, we aim to develop a simple model that is more capable than PWM and is still easy to deploy; we do not intend to compete with the best splice models which are orders of magnitude larger.

## 2. Methods

**Fig. 2.**
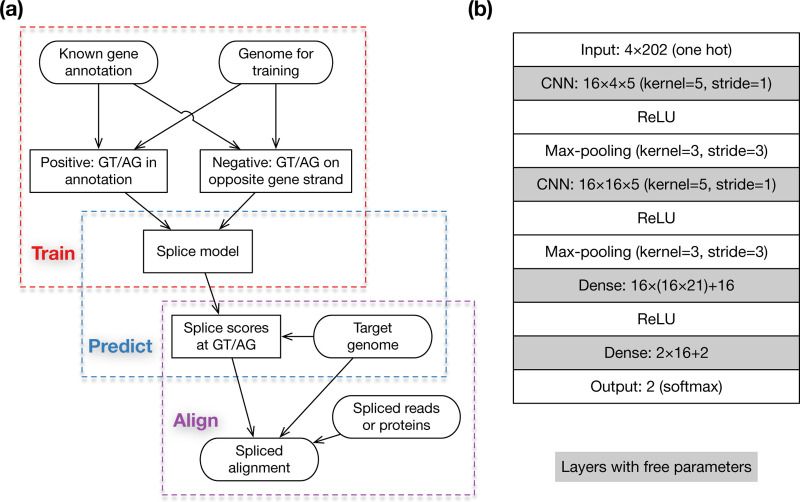
Method overview. **(a)** Overall workflow. Training and prediction are done by minisplice. Alignment is done by minimap2 or miniprot. **(b)** Default model architecture and parameterization. Shaded boxes contain free parameters.

Our overall workflow consists of three steps: training, prediction and alignment (Fig. 2a). First, we train a deep learning model and transform scores outputted by the model to empirical probabilities using known gene annotation. Second, given a target genome to which mRNA or protein sequences will be aligned, we predict the empirical probability of splice sites at each GT or AG in the genome and output the logarithm-scaled splice scores to a file. Third, when aligning mRNA sequences with minimap2 (Li, 2018) or aligning protein sequences with miniprot (Li, 2023), we feed the precomputed splice scores to the aligners which use the scores during dynamic-programming-based residue alignment. This procedure will improve the accuracy around splice sites. As we will show later, we can merge the training data from several vertebrates and insect to obtain a model working well across phyla. We do not need to train often.

### 2.1. Generating training data

Consistent with the literature, we call the 5’-end of an intron as the *donor* site and 3’-end as the *acceptor* site. Minisplice takes the genome sequence in the FASTA format and gene annotation in the 12-column BED format (BED12) as input. It provides a script to convert annotation in GTF or GFF3 format to BED12. To generate training data, minisplice inspects each annotated donor site and labels it as a positive site if its sequence is GT; similarly, minisplice labels a positive acceptor if it is an annotated AG. We ignore donor sites without GT or acceptor sites without AG because other types of splice sites are rare (Sibley et al., 2016). Miniprot and minimap2 still consider rarer GC..AG or AT..AC at the alignment step.

Due to potentially incomplete gene annotation in non-model organisms, a GT dinucleotide could be a real unannotated donor site. To alleviate this issue, we label an unannotated GT as a negative donor site only if it comes from the opposite strand of an annotated gene (Chao et al., 2024). In the rare case when two genes on opposite strands overlap with each other, we ignore the overlapping region. To balance positive and negative sites, we randomly selsect a subset of negative sites such that the positive-to-negative ratio is 1:3. For each positive or negative donor site, we extract 100bp immediately before and after GT. The total length of sequences used for training is thus 202bp. Negative acceptor sequences are generated similarly.

### 2.2. Model architecture

Minisplice uses a model with two 1D-CNN layers (Fig. 2b). The architecture is common among small models for splice site prediction (Zabardast et al., 2023). The default model uses 16 features at both CNN layers and has 7,026 free parameters in total (sum of numbers in shaded boxes). During development, we experimented alternative models, for example with different kernel sizes, more CNN or dense layers, optional dropout layers or more parameters. We chose a relatively small model in the end because it is more efficient to deploy.

### 2.3. Training and testing

We use 80% of genes on the odd chromosomes or contigs for training and reserve the rest 20% for validation. We stop training if the validation cost increases over several epochs. Recall that we intend to predict splice sites across the whole genome but when generating training data, we downsample negative GT/AG to a small fraction. To test the model accuracy in a setting closer to the prediction task, we apply the trained model to every GT/AG on the even chromosomes or contigs and compare the prediction to the known gene annotation to measure accuracy. In comparison to training data, testing data may contain errors in known gene annotation: missing junctions in the annotation would appear to be false positives (FPs), while falsely annotated junctions would look false negatives (FNs). It is not straightforward to compare accuracy across species.

### 2.4. Transforming raw model scores to probabilities

With the ‘softmax’ operator at the end, the model scores each candidate splice site with a number between 0 and 1, the higher the better. This score is not a probability in particular when the property of the training data is distinct from our intended application. We need to transform this score to probability to work with the probability-based scoring system of miniprot.

We evenly divide raw model scores into b bins (50 by default) such that raw score t∈[0,1) is assigned to bin $i=\left\lfloor tb\right\rfloor$, i=0,1,…,b−1. Let $P_{i}$ be the number of annotated splice sites scored to bin i and $N_{i}$ be the number of unannotated GT/AG sites scored to bin i. $P_{i}/\left(P_{i}+N_{i}\right)$ is the empirical probability of a candidate site in bin i being real. Let $P=\sum_{i}P_{i}$ and $N=\sum_{i}N_{i}$. Given raw score t, the transformed score is

(1) $$s(t)≜2\log_{2}\left(\frac{P_{\left\lfloor tb\right\rfloor }}{P_{\left\lfloor tb\right\rfloor }+N_{\left\lfloor tb\right\rfloor }}\cdot \frac{P+N}{P}\right)$$

It computes the log odds of the probability estimated with the deep learning model versus with the null model that assumes every GT/AG having equal probability of being real. The 2 log_2_ scaling is imposed by BLOSUM scoring matrices (Henikoff and Henikoff, 1992) which miniprot uses.

### 2.5. Aligner integration

Minimap2 (Li, 2018) uses the following equation for spliced alignment:

(2) $$\left\{\begin{array}{l} H_{ij}=\max\left\{H_{i-1,j-1}+s(i,j),E_{ij},F_{ij},{\tilde{E}}_{ij}-a(i)\right\}\\ E_{i+1,j}=\max\left\{H_{ij}-q,E_{ij}\right\}-e\\ F_{i,j+1}=\max\left\{H_{ij}-q,F_{ij}\right\}-e\\ {\tilde{E}}_{i+1,j}=\max\left\{H_{ij}-d(i)-\tilde{q},{\tilde{E}}_{ij}\right\} \end{array}\right.$$

where q is the gap open penalty, e the gap extension penalty and s(i,j) gives the substitution score between the i-th position on the reference and the j-th position on the query sequence. d(i)and a(i) are the donor and acceptor scores, respectively, calculated with Eq. (1). Miniprot (Li, 2023) uses a more complex equation which has the same donor and acceptor score functions.

### 2.6. Implementation

Minisplice is implemented in the C programming language with the only dependency being zlib for reading compressed files. It uses a deep-learning library we developed earlier for identifying human centromeric repeats (Li, 2019). Minisplice outputs splice scores in a TAB-delimited format like:

**Table:** 

chr2	4184146	+	A	9
chr2	4184167	+	A	−5
chr2	4184191	-	D	5
chr2	4184199	+	A	−5
chr2	4184213	+	D	3
chr2	4184219	-	A	1

where the second column corresponds to the offset of the splice boundary and the last column gives the splice score. We modified minimap2 and miniprot to optionally take such a file as input and use the splice scores during residue alignment. Notably, minimap2 and miniprot do not directly depend on minisplice. Users can provide splice scores estimated by other means in principle.

## 3. Results

We evaluated the accuracy of trained models using Receiver Operating Characteristic (ROC) curves where we computed the true positive rate and false positive rate at different thresholds on raw model scores. To find a small model that is generalized to multiple species and is fast to deploy, we experimented models under several settings. Our final model is trained on six insect genomes from five orders and seven vertebrate genomes. We did not train a plant model because we are less familiar with the plant phylogeny.

### 3.1. Datasets

We acquired the genome sequences and gene annotations for six model organisms and 16 non-model organisms (Table 1). For model organisms, only chicken is annotated with the Ensembl pipeline; others are annotated by third parties. For non-model organisms, we intentionally chose species that have PacBio HiFi assemblies and are annotated by both RefSeq and Ensembl if possible. Only aDenEbr and sMobHyp do not have Ensembl annotations.

### 3.2. Intra-species training

**Fig. 3.**
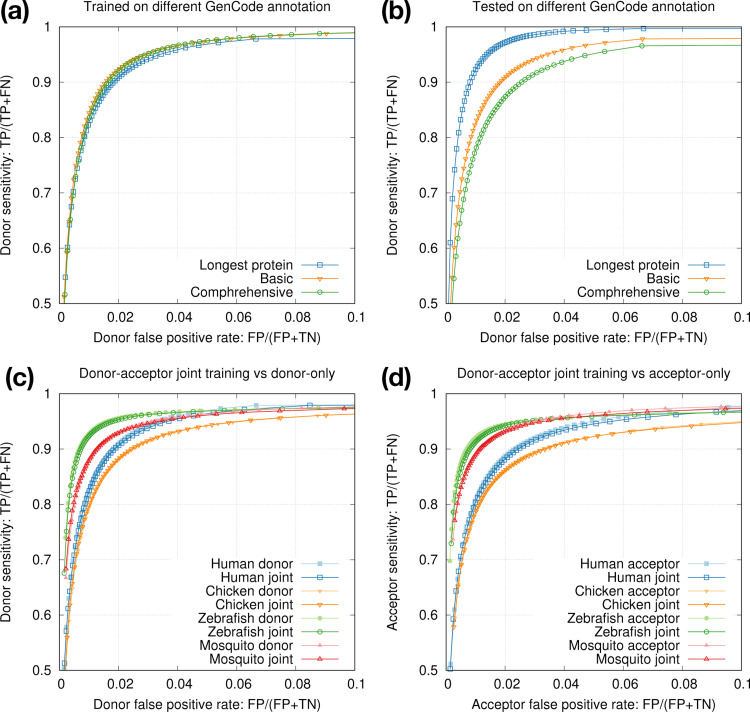
Intra-species training. **(a)** Models trained on different human annotations and tested on the Gencode basic annotation. **(b)** Model trained from the longest human protein-coding transcripts and tested on different human annotations. **(c)** Donor-only training versus joint donor-acceptor training. **(d)** Acceptor-only training versus joint donor-acceptor training.

Gencode provides a smaller set of “basic” gene annotations and a larger set of “comprehensive” annotations. We also have an option to select the longest transcript of each protein-coding gene for high-fidelity splice sites. We found training data had minor effect on testing accuracy (Fig. 3a) but annotations used for testing had larger effect (Fig. 3b). We decided to train on splice sites from the longest protein-coding transcripts for training as they are most accurate, and to test on basic annotations because non-model organisms probably do not have annotations comparable to comprehensive Gencode annotations.

The two central bases in donor training data are always GT and the two bases in acceptor training data are always AG. We speculated 1D-CNN models could easily learn the difference, so we mixed donor and acceptor training data and trained one joint model for each species. The joint models achieved nearly the same accuracy as separate donor or acceptor models (Fig. 3c,d). In later experiments, we thus always trained one joint model to simplify the training process.

### 3.3. Cross-species training

**Fig. 4.**
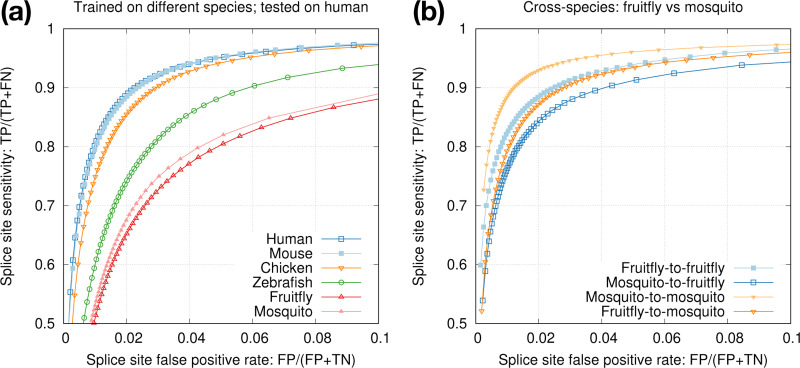
Cross-species training. **(a)** Models trained from different species and tested on human. **(b)** Intra- versus cross-species training with mosquito and fruitfly.

Our end goal is to improve spliced alignment accuracy for species without known annotations. Training and prediction are often applied to different species. To test how well a model trained from one species can predict splice sites in a different species, we applied models trained from model organisms to human (Fig. 4a). We can see the test accuracy drops quickly with increased evolution distance. The mouse model is almost good as the human model because mammals are closely related. We also applied the mosquito model to the fruitfly genome (Fig. 4b). The test accuracy is lower than the accuracy we obtain with the fruitfly model, but the drop is much smaller in comparison to applying the mosquito model to human. These experiments suggested we can achieve reasonable accuracy with a model trained from a closely related species.

**Fig. 5.**
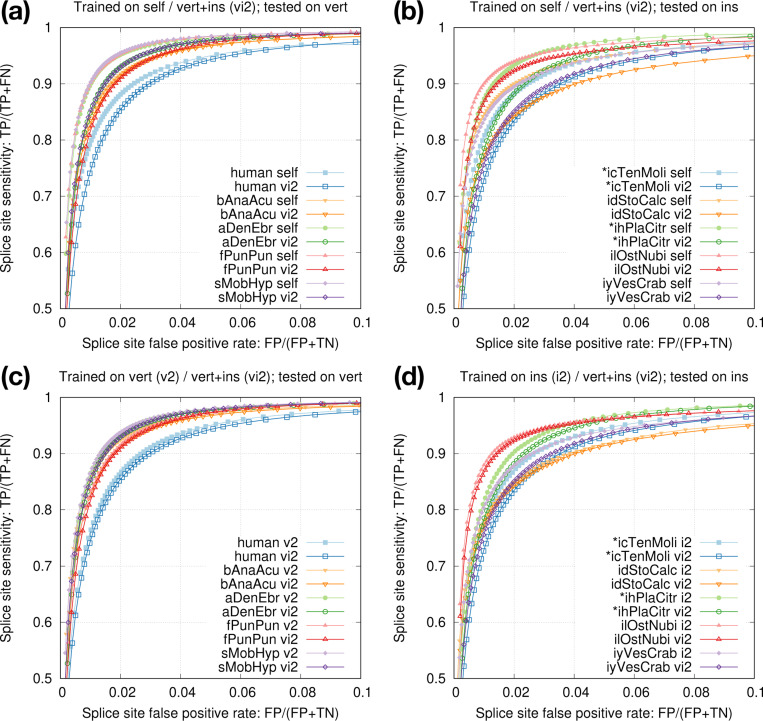
Training from multiple species. **(a)** Accuracy of model vi2 on vertebrate genomes. vi2 is trained from six vertebrate and seven insect genomes. **(b)** Accuracy of vi2 on insect genomes. Odd chromosomes in starred species are used for training. Testing is applied to even chromosomes only. **(c)** Comparison between vi2 and a model trained from vertebrate genomes (v2). **(d)** Comparison between vi2 and a model trained from insect genomes (i2).

We went a step further by combining the training data across multiple vertebrate and insect species and derived model vi2, which was trained from six vertebrate and seven insect genomes marked in Table 1. Although this model is not as accurate as the species-specific model trained from individual species itself (Fig. 5a,b), it is better than applying an insect model to human (Fig. 4a). Note that vi2 is not trained on amphibian or shark genomes but it still accurately predicts splice sites in aDenEbr and sMobHyp. It is capturing common signals across large evolutionary distance while reducing overfitting to individual species. We also trained a vertebrate-only model (v2) and an insect-only model (i2). They outperformed vi2 for vertebrate and insect genomes, respectively (Fig. 5c,d), but we deemed the improvement is marginal and outweighed by the convenience of having one model across vertebrate and insect genomes.

**Fig. 6.**
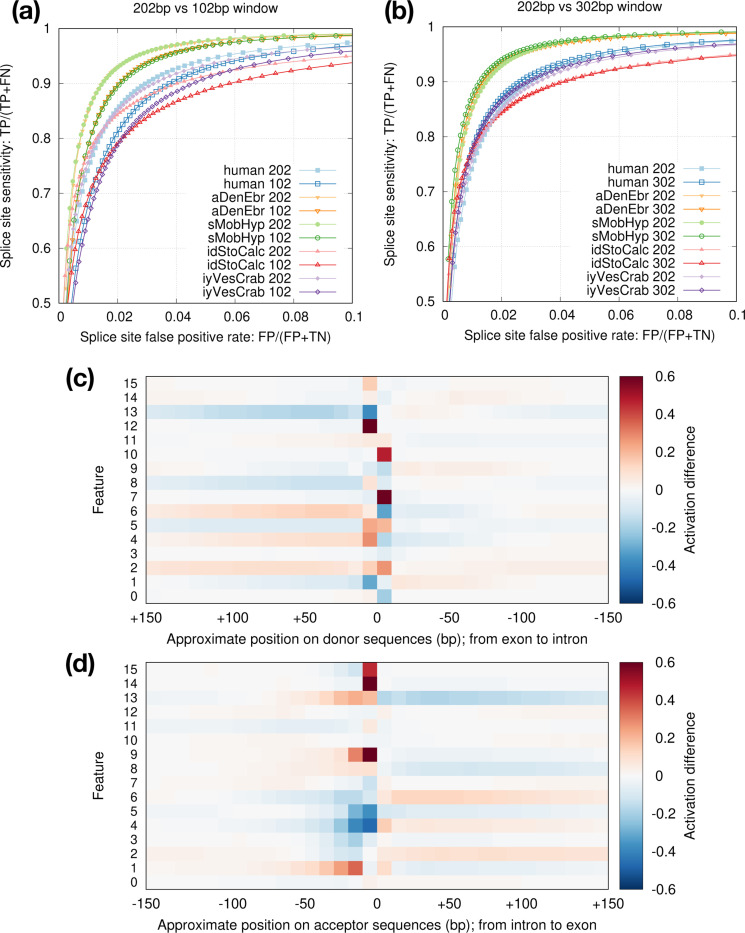
Effect of window size. **(a)** Comparison between the default 202bp window size (*±*100bp around GT/AG) and 102bp. **(b)** Comparison between 202bp and 302bp window sizes. **(c)** Activation differences between positive and negative donor sites at the last max-pooling layer. With 302bp window, this layer consists of 16 *×* 32 cells which are non-negative due to the ReLU function. Given a set of sequences, the activation rate of a cell is the frequency of the cell being positive (i.e. non-zero). The heat shows the rate difference between positive and negative sets. Dark red indicates more frequent activation among positive donor sites; dark blue is the opposite. **(d)** Activation difference for acceptor sites.

Model vi2 considers 202bp sequences around splice sites. We tried to reduce the window size to 102bp but the accuracy noticeably dropped (Fig. 6a). Increasing the window size to 302bp, on the other hand, only had minor effect (Fig. 6b). To understand what signals the model is learning, we visualized the activation rates at the last max-pooling layer which has 16 features (Fig. 6c,d). While some features such as 4 and 7 focus on signals at splice sites, other features such as 2 and 13 likely capture compositions of introns and exons. Intronic sequences around acceptors appear to provide more signals than around donors. This might be related to branch points which are located within tens of basepairs upstream to acceptors. The activation difference fades away beyond 100bp around splice sites especially within introns. It might explain why increasing window size from 202bp to 302bp has minor effect (Fig. 6b).

**Fig. 7.**
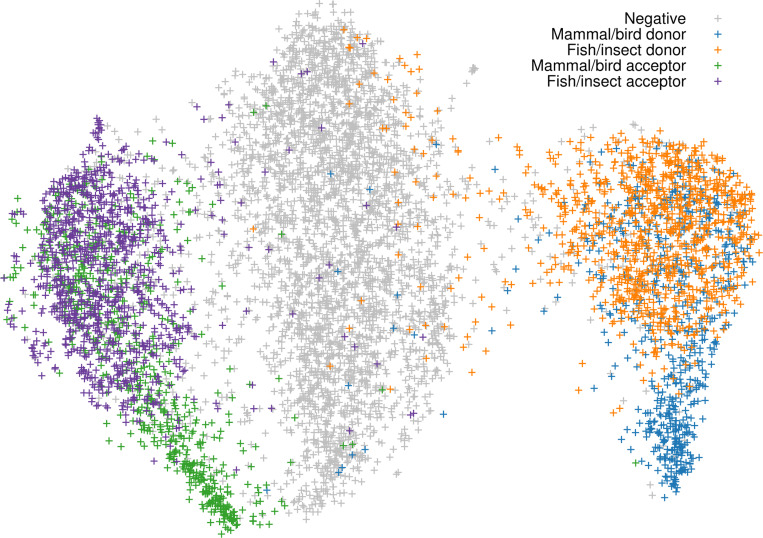
UMAP of the last max-pooling layer. Training data was downsampled to 20k splice sites with 10k positive and 10k negative sites.

We further used the values at the last max-pooling layer to generate the UMAP of a random subset of training samples (Fig. 7). The UMAP separated the training samples into three large clusters which correspond to positive acceptor sites, negative sites and positive donor sites, respectively. Notably, mammals and birds contain sites absent from fish and insect. The UMAP for human, zebrafish and fruitly reveals a similar pattern. We then extracted splice sites in the UMAP that are specific to human. We found most of the intron sequences close to the splice sites tended to be short and GC-rich. We randomly selected five human genes with GC-rich donor sites, including *GAPDH*, *ARHGEF19*, *ABTB2*, *CCAR2* and *OGDH*, and aligned their protein sequences to the zebrafish genome. Except *CCAR2* which was aligned with low identity at 33%, other genes all reserved the GC-rich junctions, though the corresponding zebrafish junctions had lower GC.

GC-rich introns are known to exist in human (Wang and Yu, 2011) and may help stabilize RNA secondary structure (Zhang et al., 2011). Their specificity to mammals and birds might explain why fish and insect models do not work well for mammals (Fig. 4a) but mammalian models tend to work better for non-mammals (McCue and Burge, 2024).

### 3.4. Improving spliced alignment

To evaluate the effect of splice models, we aligned zebrafish proteins to the human genome with miniprot (Table 2) and checked if an aligned junction is annotated in human. Unannotated junctions are likely errors in the protein-to-genome alignment. If we ask miniprot to only look for the minimal GT..AG signal, 14.01% of aligned junctions are not annotated in human. This percentage drops with improved splice models and reaches 4.37% when minisplice scores are considered during alignment. The similar trend is also observed when aligning mosquito proteins to the fruitfly genome. This confirms the critical role of splice models in protein-to-genome alignment.

**Fig. 8.**
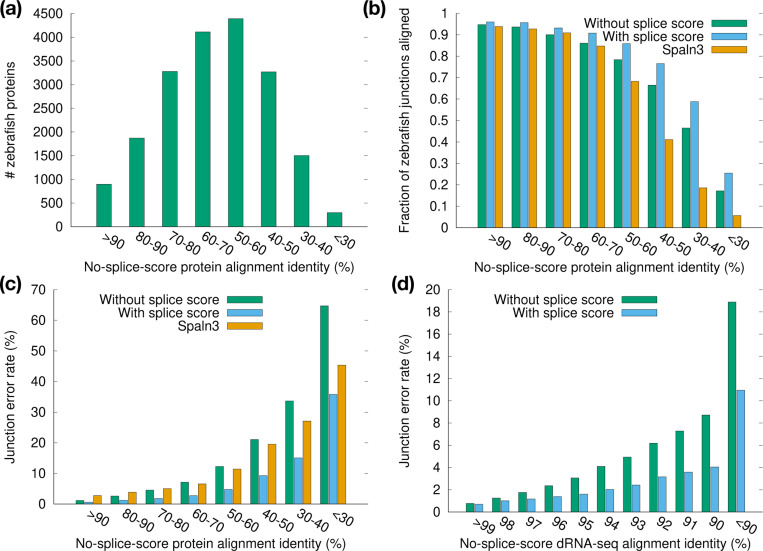
Junction accuracy stratified by alignment identity. **(a)** Number of zebrafish proteins in each identity bin. Identity is estimated from alignment of zebrafish proteins against the human genome under the default miniprot splice model. **(b)** Number of aligned junctions divided by the number of coding junctions in the zebrafish annotation. **(c)** Junction error rate of protein-to-genome alignment. **(d)** Junction error rate of RNA-seq alignment. HG002 direct-RNA reads are aligned to the human genome with minimap2.

We stratified the zebrafish proteins by their alignment identity (Fig. 8a) and inspected the alignment accuracy in each identity bin. The fraction of aligned junctions and the junction accuracy both drop with alignment identity (Fig. 8b,c). The minisplice model achieves higher accuracy across all bins, often halving the error rate in comparison to the default miniprot model.

The minisplice model also greatly reduces the junction error rate for direct RNA-seq reads (Fig. 8d), though the reduction is less pronounced when the alignment identity reaches >98%. For this RNA-seq run with the latest the Nanopore RNA004 kit (Zheng et al., 2025), 74.6% of reads are mapped with identity >98%. The overall junction error rate is marginally reduced from 1.4% to 1.0%. The advantage of advanced splice models will be more noticeable for old data of lower quality, regions of high diversity or cross-species cDNA-to-genome alignment.

## 4. Discussions

Minisplice is a command-line tool to learn splice signals with 1D-CNN and to predict the probability of splicing in the whole genome. While neural networks have long been applied to the modeling of splice sites (Reese et al., 1997), minisplice represents the first effort to directly integrate deep learning into alignment algorithms. The integration substantially improves the splice junction accuracy of minimap2 and miniprot, and is likely to help downstream analyses such as transcriptome reconstruction and gene annotation.

A marked limitation of minisplice is that it only models GT..AG splice sites. Although miniprot considers GC..AG and AT..AC signals and minimap2 has recently adopted the same model, they may still prefer GT..AG well scored by minisplice and misalign introns with non-GT..AG splicing. In addition, minisplice predicts splice scores from the reference genome. It is not aware of mutations in other samples that may alter splice signals. This bias towards the reference genome may lead to misalignment in rare cases but it is minor in comparison to annotation-guided alignment implemented in minimap2, which is biased towards both the reference genome and the reference annotation.

If we could predict splice sites to 100% accuracy, we would be close to solving the *ab initio* gene finding problem. However, the conserved splice signals only come from several bases around splice sites. Given hundreds of millions of GT/AG in a mammalian genome, we would make many wrong predictions just based on the several bases. The activation pattern at an internal layer suggests our model draws power from long-range composition of exonic sequences in addition to short-range signals around splice sites. The model may tend to classify a site as donor if the sequence on the left is similar to exonic sequences in composition. With ≥10kb windows and orders of magnitude more parameters, recent deep learning models such as SpliceAI, Pangolin and DeltaSplice will learn composition better. They may additionally see the promoter regions of many genes and species- or even tissue-specific regulatory elements. It is not clear how much their signals come from splice sites. At this point, methods combining HMM and deep learning (Gabriel et al., 2024; Holst et al., 2023) may be more advantegeous for coding sequences as they explicitly model gene structures and keep protein sequences in phase.

## Figures and Tables

**Table 1.** Datasets

Label	Species	Accession
human^†^	*Homo sapiens*	GCA_000001405.29
mouse^†^*	*Mus musculus*	GCA_000001635.9
chicken^†^*	*Gallus gallus*	GCA_016699485.1
zebrafish^†^*	*Danio rerio*	GCA_000002035.4
fruitfly^†^*	*Drosophila melanogaster*	GCA_000001215.4
mosquito^†^	*Anopheles gambiae*	GCA_943734735.2
mCanLup*	*Canis lupus baileyi*	GCF_048164855.1
mLagAlb*	*Lagenorhynchus albirostris*	GCF_949774975.1
bAccGen*	*Astur gentilis*	GCF_929443795.1
bAnaAcu	*Anas acuta*	GCF_963932015.1
aDenEbr	*Dendropsophus ebraccatus*	GCF_027789765.1
fCarCar*	*Carassius carassius*	GCF_963082965.1
fPunPun	*Pungitius pungitius*	GCF_949316345.1
sMobHyp	*Mobula hypostoma*	GCF_963921235.1
icTenMoli*	*Tenebrio molitor*	GCF_963966145.1
idCalVici*	*Calliphora vicina*	GCF_958450345.1
idStoCalc	*Stomoxys calcitrans*	GCF_963082655.1
ihPlaCitr*	*Planococcus citri*	GCF_950023065.1
ilCydFagi*	*Cydia fagiglandana*	GCF_963556715.1
ilOstNubi	*Ostrinia nubilalis*	GCF_963855985.1
iyBomTerr*	*Bombus terrestris*	GCF_910591885.1
iyVesCrab	*Vespa crabro*	GCF_910589235.1

Ensembl or Gencode annotations were used for model organisms (marked by “†”); RefSeq annotations used for non-model organisms whose labels follow the naming standard developed by the Darwin Tree of Life Project: prefix “m” stands for mammals, “b” for birds, “a” for *Amphibia* (e.g. frogs and newts), “f” for fish, “s” for sharks, “ic” for order *Coleoptera* (beetles), “id” for *Diptera* (flies), “ih” for *Hemiptera* (true bugs), “il” for *Lepidoptera* (butterflies and moths), “iy” for *Hymenoptera* (bees and ants). Species marked by “*” are used for training cross-species models.

**Table 2.** Effect of splice site models on protein-to-genome alignment

	zebrafish proteins to human genome					mosquito proteins to fruitfly genome				
Aligner Splice model	miniprot GT-AG	miniprot default	miniprot extended	miniprot minisplice	Spaln3 Tetrapod	miniprot GT-AG	miniprot default	miniprot extended	miniprot minisplice	Spaln3 InsectDm
# prediced junc	168,030	164,094	160,935	164,860	144,544	30,279	28,780	27,307	28,722	24,538
# annotated junc	144,495	146,898	147,880	157,654	132,394	24,022	24,465	24,203	27,107	21,161
% unannotated	14.01	10.48	8.11	4.37	8.41	20.68	14.99	11.37	5.62	13.76
% Base Sn	60.09	60.12	60.04	60.85	51.71	57.00	56.92	56.76	57.28	44.53
% Base Sp	94.33	95.49	96.09	97.30	94.37	98.39	98.65	98.64	99.28	97.37

For the splice models, “GT-AG” only considers GT..AG, “default” considers GTR..YAG, “extended” additionally considers G|GTR..YYYAG, and “minisplice” uses the “vi2–7k” model on top of “extended”. Miniprot was invoked with “-I” which sets the maximum intron length to $\min\left\{\max\left\{3.6\sqrt{G},10^{4}\right\},3\times 10^{5}\right\}$, where G is the genome size. Spaln3 was invoked with “-LS -yS -yB -yZ -yX2” and with its own splice models.

% unannotated = 1 –

# annotated/

# predicted.

## Data Availability

The minisplice source code is available at https://github.com/lh3/minisplice. Pretrained models can be obtained from https://doi.org/10.5281/zenodo.15446314.
